# Madness and society in Britain

**DOI:** 10.1192/bjb.2022.45

**Published:** 2023-06

**Authors:** George Ikkos, Nick Bouras, Peter Tyrer

**Affiliations:** 1 Royal National Orthopaedic Hospital NHS Trust, Stanmore, UK; 2 King's College London, UK; 3 Imperial College, London, UK; 4 Lincolnshire Partnership NHS Foundation Trust, Lincoln, UK

**Keywords:** History of psychiatry, deinstitutionalisation and community care, meta-community psychiatry and mental healthcare, mental health awareness

## Abstract

The fiftieth anniversary of the Royal College of Psychiatrists, and the publication of a detailed multidisciplinary social history of British psychiatry and mental health in recent decades have offered an opportunity to take a helicopter view and reflect on the relation between psychiatry and changing British society. We argue that the time has come to move on from the rhetoric of deinstitutionalisation and community mental healthcare to lead public debate and advocacy for the needs of the mentally ill in the new era of ‘meta-community psychiatry and mental healthcare’. We need to respond effectively to the increasing awareness of mental health problems across society, aiming for a pluralist, integrated and well-funded reform led by joint professional and patient interests which could be unstoppable if we all work together.

Anthony Ashley Cooper, 7th Earl of Shaftesbury, was an Evangelical Christian who believed it is one's duty to help the least fortunate in society. He was at the vanguard of parliamentary legislation, which from 1845 onwards mandated the creation of a regulated countrywide mental asylum system. This was significantly inspired by The Retreat, a model institution set up by the Quakers for their distressed members. The assumption was that through ‘moral treatment’ mental asylums would be therapeutic. Treatment approaches changed over the years but, after a period of optimism and energy in the later 19th century, there was a process of passive accumulation that led by the mid-1950s to 150 000 people living in what since 1930 had been called mental hospitals in Britain. Yet all had begun to change.

The publication of the open-access volume *Mind, State and Society: Social History of Psychiatry and Mental Health in Britain 1960–2010*,^1^ on the 50th anniversary of the Supplemental Charter that gave the Royal Medico-Psychological Association the status of the Royal College of Psychiatrists, offers an opportunity to reflect on the relation between psychiatry and our rapidly changing British society in recent decades and to set orientation for the future. We aim to look at the broad sweep of change. Has the transition from asylum to community in adult mental healthcare fulfilled its ambitions? What have been some key people, policies and events that have shaped outcomes? How has the profession responded? And could we do better?

We propose that it is time to move on from the worn and tired rhetoric of community psychiatry to develop new thinking: ‘meta-community psychiatry and mental healthcare’. In Greek ‘meta’ means after. So, we refer to what comes ‘after community psychiatry and mental healthcare’.

## Deinstitutionalisation and community care

The damning critiques of the sociologist Erving Goffman, social theorist Michel Foucault and radical psychiatrist Thomas Szasz marked the end of faith in mental hospitals as therapeutic institutions. Building on changes heralded by progress in psychopharmacology and changes in mental health law in the 1950s, the Conservative Minister for Health Enoch Powell accelerated the course of their forthcoming demise in 1961 with his often quoted ‘water tower speech’, delivered in his careful and deliberate Birmingham accent. Powell was educated close to Hollymoor Hospital at King Edward's School in Birmingham, and cycling to school each day he would have passed Hollymoor's water tower, the largest in the country, with its imposing green copper dome. So, when he referred to asylums as standing ‘isolated, majestic, imperious, brooded over by the gigantic water tower and chimney combined’ he may have had Hollymoor in mind. In adding ‘do not for a moment underestimate their power of resistance to our assault’, he was reflecting on the apparent permanence of these buildings. He was right. This impressive listed building is still standing.

Powell set in motion the abolition of the mental hospitals. People with acute mental health problems were now meant to be looked after in district general hospital units. During the next two decades, mental hospital numbers began to decline; they were underfunded and disrespected, and residents suffered accordingly. By the late 1980s bed numbers had halved but the hospitals themselves still stood. It was not until Margaret Thatcher's National Health Service and Community Care Act 1990 that their physical demise was sealed. Although both Powell and Thatcher were Conservative politicians, they enjoyed widespread support across the political spectrum regarding the closing of the old asylums. Deinstitutionalisation and community care in mental health materialised during socially liberal and economically neoliberal times.

Since their inception, mental health services have often experienced, and reforms been driven by, underfunding and scandals. Community psychiatry proved no exception, especially as it was hit by the financial fallout from the 1973 oil crisis and the decline of the welfare state. The welfare state had lain at the heart of its assumptions. The period covered by *Mind, State and Society* is one that could be summarised as the growth and exploitation of community psychiatry. The growth started organically; psychiatrists and other health professionals who were dissatisfied with stasis and the stultifying lack of progress in mental hospitals moved their work increasingly into the community in the forms available at the time, mainly out-patient clinics, day hospitals and rehabilitation settings. In some countries, notably Italy, this even became a political movement and led to the rapid closure of mental hospitals. In the UK, change was more gradual but was effective in reducing bed usage greatly. Some enthusiasts went further and expanded services into primary care, again with further reduction of beds.^2^ A healthy balance was achieved between hospital provision and community care, and more services moved out of both mental hospitals and psychiatric units in general hospitals.

Then governments of all persuasions, always keen to promote any change that looked as though it saved money, jumped enthusiastically on the community bandwagon and promoted the closure of beds from the 1980s onwards, especially since 1990. This accelerated later, to the extent that between 1998 and 2014 the UK reduced its psychiatric bed complement from 100 beds per 100 000 to 45 per 100 000, faster than any other OECD country.^3^ Many initiatives to promote even more services to reduce the need for beds were supported – these even extended to prisons. This was the phase of exploitation. The mental health services were exploited by other government departments, which did not take the advice of the experts who, in pointing out the considerable income being generated from hospital closures, emphasised that the savings should be kept within psychiatric services and not diverted elsewhere. The recommendation that ‘service providers and purchasers should focus on developing community-based care (including increased provision of 24 h nursed beds) by ensuring that resources released through earlier closure programmes have been redeployed for their intended use’ was never followed.^4^

The consequences of this exploitation have not been positive. In-patient wards are a chaotic travesty of the calm and reflective environments that the Victorian planners had aspired to and sometimes created, community teams have been fragmented into different elements that lack any form of integration and the notion of continuity of care has become a running joke. We consider the response of the Royal College of Psychiatrists to identification of failures^5^ and cries of alarm^6^ to have been tentative and ineffective as judged by outcomes,^7^ although others take a more sanguine view with respect to progress.^8^ The College's efforts have not been helped of course by very few Ministers for Health having any understanding of mental illness, partly because of the lack of impact it has on voting patterns. This is a problem when government in the UK is the dominant service provider as well as policy maker and, ultimately, a vital source of funds for the College's projects.

## Social inclusion and exclusion

One welcome development of a new consumerist approach to (mental) health services was the greater voice of service users and their advocacy organisations, especially MIND, which started life as the National Association for Mental Health, and whose chair, Kenneth Robinson, subsequently became a Labour Minister for Health (1964–1968). But while the abolition of mental hospitals was popular, community care was much less so. Many people did not like people they did not understand living on their doorstep. Troubles mounted. Not only were hospital beds lost but rehabilitation units and hostels for the homeless too. Homeless hostels had hitherto functioned as a parallel mental health service for many, particularly those with addictions. This happened at the same time as Conservative governments restricted housing and other benefits available to the young, including during periods of high unemployment. The impact on the care of mentally ill people has been devastating, with revolving door clinical care policies causing street destitution and trans-institutionalisation to prisons.

Vital human rights and deinstitutionalisation rhetoric aside, a new national network of medium secure units for people with mental health problems arose, and the number of people detained involuntarily gradually began increasing. Urged by sensational tabloid headlines such as *The Sun*'s ‘1,200 killed by mental patients’ (7 October 2013), communities rejected rather than engaged positively with those with severe mental illness. In fact, contemporaneous statistics showed that violent offending by people with mental illness had declined by 3% per year during the decades under review here.^9^ People with mental health problems were far more likely to be the victims than perpetrators of violent acts.

To stem tabloid headlines, Tony Blair's Labour government practically invented a mental disorder which had no basis in science and the aim of which was not the treatment of mental ill health but the imagined protection of the public. Despite the opposition of all mental health professions, government, led by the Home Office rather than the Department of Health, spent £480 million on a ‘treatment programme’ for people with the invented disorder of ‘DSPD’ (dangerous and severe personality disorder). Results were predictably negative.^10^

Not least in response to the above, policies of deinstitutionalisation and community care have been associated with professional, patient and advocate efforts to destigmatise mental illness. This aim is laudable, and the welcome effect has been that we now talk more freely about ‘mental health’. However, it has become increasingly difficult to talk about ‘mental illness’. Although this may please psychiatry's most vociferous critics, it does not do away with the problem. Careful longer-term follow-up studies of patients with schizophrenia who had been resident in the old asylums found deterioration in mental and physical health after discharge to the community.^11^ Neoliberal marketisation of healthcare, leading to breakdown of continuity of care in the community, has had demonstrably negative effects on clinical outcomes.^12^

## Present state examination

Since the 1960s, Britain has been rocked by regular political, economic and moral crises. Nevertheless, adjusted for inflation, national wealth has grown more than threefold (338%) and the proportion of such wealth allocated to healthcare has more than doubled, from 3.1 to 7.5%.^13^ However, although the country has become richer, inequality has increased and wealth has been inequitably distributed, with intersection of economic inequality with other inequalities (e.g. ethic, gender, age, sexual orientation). Poverty and health inequalities also increased and those disabled, particularly the severely mentally ill, have suffered the greatest inequality, with grave consequences. With mental ill health accounting for 22% of national burden of disease but only 11% of spend, compared with physical healthcare and clinical need, allocation to mental health services has been disproportionately low. Following the global financial crisis of 2008, problems have escalated, especially since the 2010 election of David Cameron's and Nick Clegg's coalition and subsequent Conservative governments’ austerity cuts and welfare reforms. Now, rates of new involuntary detentions are higher than ever before and people with schizophrenia die 10–20 years earlier than the average in the population.^14^ Not only patients but also their families have all too often been left to pick up the pieces. It is a national scandal. The implications of the COVID-19 pandemic to the mental health of citizens globally are expected to add further colossal problems. The invasion of Ukraine and its aftermath too.

At the same time as policies of deinstitutionalisation and community care were being introduced and implemented, communities were weakening. This has been captured vividly by contemporary politicians in telling phrases such as ‘there is no such thing as society’ (Margaret Thatcher), ‘get on your bike’ (Norman Tebbit) and ‘citizens of nowhere’ (Theresa May). Crucial have been the rise of IT, personal computing and social media and the consequent emergence of something profoundly different, namely digital communities. As explored in detail by David Edgerton in *The Rise and Fall of the British Nation*,^15^ even the national ‘community’ did not hold together. If we aim to build a better society after the neoliberal crisis and COVID-19, we must move forward from the tired and increasingly mistrusted rhetoric of community psychiatry and mental healthcare.

As noted above, in Greek ‘meta’ means after. If we are to do justice to madness as well as mental health, we must imagine and utilise new ideas and new tools to provide effective, collaborative and genuinely pluralist ‘meta-community’ approaches. These must embrace actively a variety of perspectives and be appropriately medically supported and informed while not inappropriately medically dominated.^16,17^ In this brief paper we intend to provide a jolt, not a blueprint. Johnson et al offer a range of helpful suggestions.^18^ However, although attention to ‘illness’ and ‘services’ must be vigilant, such focus remains too narrow. There is a need to complement aspirations to excellence in medicine and practical understanding of neuroscience and health services management with deeper training in the social sciences and serious and persistent engagement with ‘mad studies’.^19^ We are not suggesting the premature abandonment of diagnoses such as schizophrenia, nor the proscription of ECT, but unless we robustly widen our professional formation and continuing education and training, we consider it unlikely that the profession will be able to respond to and work together with others to lead in the forthcoming transformations. We refer to transformation of technologies, methods of communication and identities. Also, transformation of communities, whether the latter are geographically or digitally related or imagined. These diverse and pervasive transformations will have a profound impact on the presentation of psychopathology, indeed its very definition. Past form confirms a very real risk of being left behind in their trail.

A most obvious message to be gained from the past quarter century is that while interest in mental health has become popularised, the influence of psychiatrists has paradoxically been reduced. When Sir George Godber was Chief Medical Officer throughout the 1960s he was the perfect liaison officer between all branches of the profession and the government. Throughout he was supporting the NHS dictum that the service should meet the needs of all and be free at the point of delivery. He extended this fully to mental healthcare also and took care to ensure that the closure of mental hospitals was accompanied by good accessible community care. He was helped in this greatly, as all Ministers for Health from Enoch Powell to Kenneth Robinson, irrespective of political party, supported this policy. In contrast, his successor, William Yellowlees (1973–1984), has been assessed in damning terms by the relevant minister of state.^20^

Since 1990 the system has splintered into dissociative chaos, with the assets of mental hospitals sold to support medical services elsewhere, divorce of forensic services into separate enclaves, unconstructive arguments about power and control in community services, downgrading of expert experience and creeping privatisation of care when bed reduction became excessive. During the decades under review, more so since the oil crisis in the 1970s and the neoliberal reforms of the UK state since the 1980s, the profession has been on the back foot, trying to keep up with government diktats based only on political whim or crude cost-cutting policies, with hapless managers having rings run round them by their well-funded private equivalents (described perfectly by former Conservative Minister Kenneth Clarke^21^).

## A note of optimism: building a realistic future

The one big positive that can promote optimism for the future is the strong chance of reform following intelligent use of personal experience of mental illness. This needs to be harnessed nationally, as in countries such as The Netherlands,^22^ so that the voice of patients is heard clearly at the highest levels of the NHS, the government and the Treasury (the last being the most important). Lobbies are often derided, but one as large as the 20% of the population who encounter mental health services can never be ignored. If this force could be engaged in genuine partnership, it would surely carry the day. To achieve this, we need to be robust about the limits as well as extent of our expertise, develop more nuanced appreciation of the relevance of social change and the sciences that make sense of it, enhance familiarity with mad studies, and engage more deeply and cooperatively with patients and other mental healthcare providers. The recent belated recognition of structural inequalities and their introduction in the curriculum are welcome but not enough. As we have argued elsewhere, the understanding of ideology, i.e. aspirations, is crucial for the effective engagement of psychiatrists with change, just as important as the understanding of ‘evidence’.^23^ We will fail in any endeavour to build equitable and effective alliances with patients unless we clearly engage with this. We would add here that understanding and engaging consistently and constructively with the ideology of other professions is just as important as understanding and engaging with patients. Understanding our own ideology too!

## About the authors

**George Ikkos** is a consultant liaison psychiatrist with Royal National Orthopaedic Hospital NHS Trust, Stanmore, UK. **Nick Bouras** is Emeritus Professor of Psychiatry at the Institute of Psychiatry, Psychology and Neuroscience, King's College London, UK. **Peter Tyrer** is Emeritus Professor of Community Psychiatry at Imperial College, London, and a consultant in transformation psychiatry with Lincolnshire Partnership NHS Foundation Trust, Lincoln, UK.

## Data availability

Data availability is not applicable to this article as no new data were created or analysed in this study.
